# The Correlation between Preoperative Average Heart Rate and Postoperative Recurrence in Patients with Paroxysmal Atrial Fibrillation Undergoing Transcatheter Radiofrequency Ablation

**DOI:** 10.31083/j.rcm2511394

**Published:** 2024-11-06

**Authors:** Xiu Feng, Ling Yang, Zhenni Yang, Yuxia Miao, Mingxia Gong, Jun Meng, Min Xu

**Affiliations:** ^1^Department of Echocardiography and Cardiology, The Third Affiliated Hospital of Soochow University, 213000 Changzhou, Jiangsu, China; ^2^Department of Cardiology, The Third Affiliated Hospital of Soochow University, 213000 Changzhou, Jiangsu, China

**Keywords:** paroxysmal atrial fibrillation, transcatheter radiofrequency ablation, postoperative recurrence, average heart rate, echocardiography

## Abstract

**Background:**

Atrial fibrillation (AF) is the most common persistent arrhythmia, with increasing incidence worldwide. Transcatheter radiofrequency ablation (RFA) represents a first-line therapy for paroxysmal atrial fibrillation (PAF), although the long-term recurrence rate of AF remains relatively high. This study aimed to investigate the relationship between the average heart rate (AHR) on a dynamic electrocardiogram before transcatheter RFA and the postoperative recurrence of AF in patients with PAF.

**Methods:**

A retrospective analysis was conducted on patients with PAF who experienced primary transcatheter RFA. Relevant clinical indicators, dynamic electrocardiograms, and echocardiography were collected from the enrolled patients before ablation. Multivariate logistic regression analysis examined the relationship between the preoperative AHR and postoperative recurrence of AF in patients with PAF.

**Results:**

This study included 224 patients with PAF who were scheduled for transcatheter RFA. Based on the AHR in sinus rhythm state on the dynamic electrocardiogram before ablation, the patients were divided into three groups: low, medium, and high heart rate. The recurrence rates of AF after ablation for the three groups were 14.667%, 8.108%, and 4.000%, respectively. After adjusting for confounding factors, postoperative AF recurrence risk gradually decreased with an increase in preoperative AHR (odds ratio: 0.849, 95% confidence interval: 0.729–0.988, *p* = 0.035). This trend remained statistically significant even after adjusting for the three categorical variables of AHR (odds ratio = 0.025, 95% confidence interval: 0.001–0.742, *p* = 0.033). The curve fitting analysis indicated a linear and negative correlation between the preoperative AHR and postoperative AF recurrence risk in patients with PAF.

**Conclusions:**

In patients with PAF who experienced their primary transcatheter RFA, there was a linear and negative correlation between the AHR in sinus rhythm state on the preoperative dynamic electrocardiogram and the risk of postoperative AF recurrence.

## 1. Introduction

Atrial fibrillation (AF) is a prevalent tachyarrhythmia in clinical practice, with rapidly increasing incidence and prevalence rates [1]. AF is associated with elevated risks of thromboembolism, heart failure, and other cardiovascular events, leading to higher mortality and disability rates [2]. AF treatment strategies include rhythm and heart rate control therapy, which are crucial components in comprehensive treatment approaches [3]. Some scholars stated there was a probability of spontaneous conversion to sinus rhythm in AF patients, so a score was developed and validated to determine the probability in patients with hemodynamically stable, symptomatic, and recent-onset AF [4]. However, a few patients with AF can convert to sinus rhythm spontaneously and have a high possibility of recurring and maintaining AF rhythm, meaning transcatheter radiofrequency ablation (RFA) has emerged as a significant treatment option for controlling rhythm, alleviating symptoms and enhancing the quality of life in patients with paroxysmal atrial fibrillation (PAF) (Ia) [5]. However, literature reports show a recurrence rate of approximately 11%–30% within 1–5 years after RFA [6, 7, 8, 9]. Thus, to optimize AF treatment strategies, predicting AF recurrence after RFA is essential. Common risk factors for AF recurrence after RFA include left atrial enlargement, prolonged AF duration, and impaired left atrium storage function. Researchers have developed several predictive models to improve the accuracy of predicting AF recurrence after RFA. For instance, Mesquita *et al*. [10] constructed and validated the ATLAS score to estimate the recurrence rate of AF after RFA, and Kornej *et al*. [11] developed the APPLE score to identify patients with low, medium, and high risks of AF recurrence, Winkle *et al*. [12] created the CAAP-AF scoring system to estimate AF recurrence within 2 years after RFA and Mujović *et al*. [13] established the MB-LATER clinical score to predict very late recurrence of AF occurring >12 months. Lohrmann *et al*. [14] concluded that in patients with continuous monitoring undergoing RFA, only CHA2DS2-VASc scores ≥5 could predict higher AF recurrence, and Letsas *et al*. [15] stated that a CHA2DS2-VASc score ≥2 was an independent predictor of AF recurrence during the follow-up. Peigh *et al*. [16] and Nayak *et al*. [17] established and validated the ability of a high SCALE-CryoAF score to specifically predict the first AF recurrence over one year and very late recurrence of AF after cryoballoon ablation. Cay *et al*. [18] developed a PAT2C2H score to identify patients likely to benefit most from cryoballoon ablation of PAF and who should be monitored more closely for arrhythmia recurrence at 12 months. Jastrzębski *et al*. [19] compared six risk scores for predicting AF recurrence after cryoballoon-based ablation and developed the 0-1-2 PL score to predict AF recurrence, which was simplified without compromising accuracy. However, there remains a need for simple and practical recurrence markers in this context.

Dynamic electrocardiography is a commonly used method for detecting arrhythmias in medical practice. This technology offers several advantages: simplicity, cost-effectiveness, and non-invasiveness. Furthermore, the average ventricular rate obtained from a dynamic electrocardiogram can more accurately reflect a patient’s cardiac activity. Research has demonstrated the significance of the average ventricular rate in guiding optimal heart rate control treatment and adjusting drug dosage in patients with AF when obtained from a 24-hour dynamic electrocardiogram; moreover, it can provide comprehensive information on AF burden and the onset of AF [20]. Another study [21] has suggested that a 24-hour dynamic electrocardiogram can serve not only in diagnosing AF but also in independently correlating ST segment depression with the occurrence of AF, thereby identifying patients with AF who are either at high or low risk.

A prospective cohort study conducted in Norway based on the general population with a follow-up period averaging 20 years has reported that individuals with a higher resting heart rate (>60 bpm) have a lower risk of developing AF compared to those with a lower resting heart rate (<50 bpm) [22]. Wu *et al*. [23] found that in PAF patients over 65 years who underwent RFA, a preoperative low resting heart rate is an independent predictor of AF recurrence. In addition, another study [24] demonstrated that in patients with paroxysmal and persistent AF, a high postoperative average heart rate (AHR) is significantly correlated with a high preoperative AHR, while a high postoperative AHR is significantly associated with a reduced clinical recurrence rate of AF.

This study retrospectively examined the 24-hour dynamic electrocardiogram of patients with PAF before undergoing RFA and analyzed the correlation between the AHR in the sinus rhythm state and postoperative recurrence of AF. The objective was to provide guidance for adjusting preoperative antiarrhythmic drug (AAD) usage while also enabling early identification and targeted intervention for patients at high risk of postoperative recurrence.

## 2. Methods

### 2.1 Research Subjects

A single-center, retrospective cohort method was used in this research. Patients with PAF admitted to Changzhou First People’s Hospital between January 2017 and December 2020 and scheduled to undergo RFA for the first time were included in this study. PAF was defined as AF with a duration of 7 days or less, mostly lasting less than 24 hours, and capable of self-termination. Inclusion criteria were as follows: (1) no significant history of organic heart disease; (2) aged between 18 and 75 years; (3) absence of cardiac insufficiency in preoperative echocardiography (left ventricular ejection fraction (LVEF) ≥50%); (4) presence of clinical symptoms during the onset of AF. Exclusion criteria included: (1) patients with a history of cardiac disease, such as valve disease, congenital heart disease, or cardiomyopathy; (2) patients with cardiac insufficiency symptoms and no improvement after treatment; (3) patients who underwent ablation of AF, surgical therapy, ablation of atrioventricular node, or other ablation-requiring arrhythmias; (4) patients with liver and kidney dysfunction (estimated glomerular filtration rate <60 mL/min/1.73 m^2^, or creatinine clearance <60 mL/min) [25]; (5) patients with thyroid diseases; (6) patients with respiratory system diseases; (7) pregnant and lactating women; (8) patients with recurrent AF within 3 months after ablation; (9) patients with incomplete clinical data and lost follow-up. This study adhered to the Helsinki Declaration and received approval from the Scientific Ethics Committee of the Third Affiliated Hospital of Soochow University (2016TNo. 44).

### 2.2 Clinical Baseline Indicators

We collected various clinical baseline indicators from the patients, including age, gender, body mass index (BMI), duration of AF, blood urea nitrogen level, blood cholesterol level, atrial fibrillation thrombus risk score (CHA2DS2-VASc score), medical history (including hypertension, diabetes mellitus, hyperlipidemia, coronary heart disease, stroke or transient ischemic attack and peripheral vascular disease), lifestyle factors (such as smoking and alcohol consumption) and the usage of AAD.

### 2.3 Acquisition of Dynamic Electrocardiogram Monitoring Parameters

Before RFA, patients underwent dynamic electrocardiogram examination within 48 hours. The BI900 series dynamic electrocardiogram monitoring system (Shenzhen Boying Biomedical Instrument Technology Co., Ltd. Shenzhen, Guangdong, China) was used for dynamic electrocardiogram recording. The instrument consisted of a smaller recorder, electrodes, and a playback system. Patients were initially positioned supine, with right arm (RA) electrodes placed at the right subclavian fossa, left arm (LA) electrodes placed at the left subclavian fossa, left leg (LL) electrodes placed at the junction of the left clavicular midline, and rib arch, right leg (RL) electrodes placed at the waist or sloping shoulder at the junction of the right clavicular midline and rib arch, and V1–V6 electrodes horizontally attached to the anterior chest rib. Once the electrodes were attached, they were connected to the small recorder and secured with a strap. Patients were instructed to avoid strenuous activity and overexertion. After 24 hours, the dynamic electrocardiogram image recording was completed and fully imported for archiving. All collected electrocardiogram data were cross-analyzed by two experienced intermediate or higher-ranked electrocardiologists, and a senior electrocardiologist reviewed the analysis results. All electrocardiologists who analyzed the dynamic electrocardiogram (ECG) recordings were blinded to the procedure outcomes. The 24-hour dynamic ECG data analysis included the assessment of AHR in sinus rhythm state, total time of AF, total episodes of AF (with each episode lasting >120 seconds), and proportion of time in AF.

### 2.4 Measurement of Echocardiographic Parameters

Prior to RFA, all patients underwent transthoracic echocardiography using the Philips EPIQ 7c color Doppler ultrasound diagnostic instrument(Philips Healthcare Royal Philips Electronics, Amsterdam, the Netherlands). Patients were positioned left-lying and connected to a 12-lead electrocardiogram. The probe utilized a center frequency of 1–5 MHz and a frame rate of 50 Hz. Standard M-type, two-dimensional, and Doppler images of the sternum and apex were obtained. Two experienced physicians collected data during sinus rhythm, capturing five cardiac cycles, according to the guidelines of the American Society of Echocardiography [26], the left atrial diameter (LAD), left ventricular end-diastolic diameter (LVEDD), left ventricular end-systolic diameter (LVESD), and LVEF, which was measured by the biplane Simpson method.

### 2.5 Transcatheter RFA

The ablation catheter and stimulation catheter were inserted into the right atrium through the femoral vein and then into the left atrium by transseptal puncture. Then, the CARTO system (Biosense Webster, Inc., Irvine, CA, USA) performed three-dimensional mapping and merged with optimal three-dimensional reconstruction by computer tomography. A Thermocool SmartTouch ST or STSF (Biosense Webster, Inc., Irvine, CA, USA) was employed to deliver the radio frequency signals at a target temperature of 45 °C and a power of 40 W. The resulting local myocardial injury had a depth and range of approximately 3–4 mm. Additionally, the radiofrequency ablation path was targeted as wide antral circumferential pulmonary vein isolation (the pulmonary vein ostium). This resulted in a reduction in local myocardial voltage to less than 0.15 mV and the elimination of pulmonary vein potential.

### 2.6 Perioperative Medication and Postoperative Follow-up

Commonly employed medications for managing ventricular rate in selected patients before ablation primarily consisted of β receptor blockers, non-dihydropyridine calcium channel blockers (ND-CCB), digoxin, and certain AAD. Furthermore, the choice of AAD treatment strategy was tailored to the patient’s condition to attain the desired ventricular rate control standards: resting heart rate ≤80 bpm and heart rate <110 bpm during moderate-intensity exercise [27].

All patients were followed up with a daily electrocardiogram for three days following the ablation procedure. They were also prescribed oral anticoagulants for a minimum of two months and continued taking their previous AAD for three months. After discharge, patients were monitored with monthly electrocardiograms, and at least one 24-hour dynamic electrocardiogram examination was conducted monthly.

### 2.7 Statistical Analysis 

R (https://www.R-project.org) and EmpowerStats software (https://www.empowerstats.com, X&Y Solutions, Inc, Boston, MA, USA) were used for the statistical analysis. Measurement data that followed a normal distribution are expressed as the mean ± standard deviation ($\bar{x}$ ± s). In cases where these data had a mildly skewed distribution, the median was used. For severely skewed distribution data, these data were transformed using the Box-Cox transformation and then expressed as the mean ± standard deviation ($\bar{x}$ ± s). Inter-group comparisons were conducted using either variance analysis or the Kruskal–Wallis test. Counting data were presented as a percentage (%), and inter-group comparisons were performed using the chi-square test.

Univariate logistic regression analysis assessed the association of different factors and AF recurrence. Multivariate logistic regression analysis was employed to evaluate whether AHR was an independent risk factor for AF recurrence. Exact and asymptotic methods were applied to analyze unadjusted and adjusted estimates, respectively. If a covariate changed the estimates of AHR on AF recurrence by greater than 10% or had a significant association (*p* < 0.10) in the univariate analysis, it would be included as a potential confounding factor in the final models. A generalized additive model was applied to check if there was a non-linear relationship between AHR and AF recurrence. The model helped to discover non-linearity and decide whether a threshold effect existed. A value of *p* < 0.05 indicated a statistically significant difference.

## 3. Results

### 3.1 Comparison of Clinical Parameters, Dynamic Electrocardiogram, and Echocardiographic Indicators with Different AHR Levels in PAF Patients

**Fig. 1.**
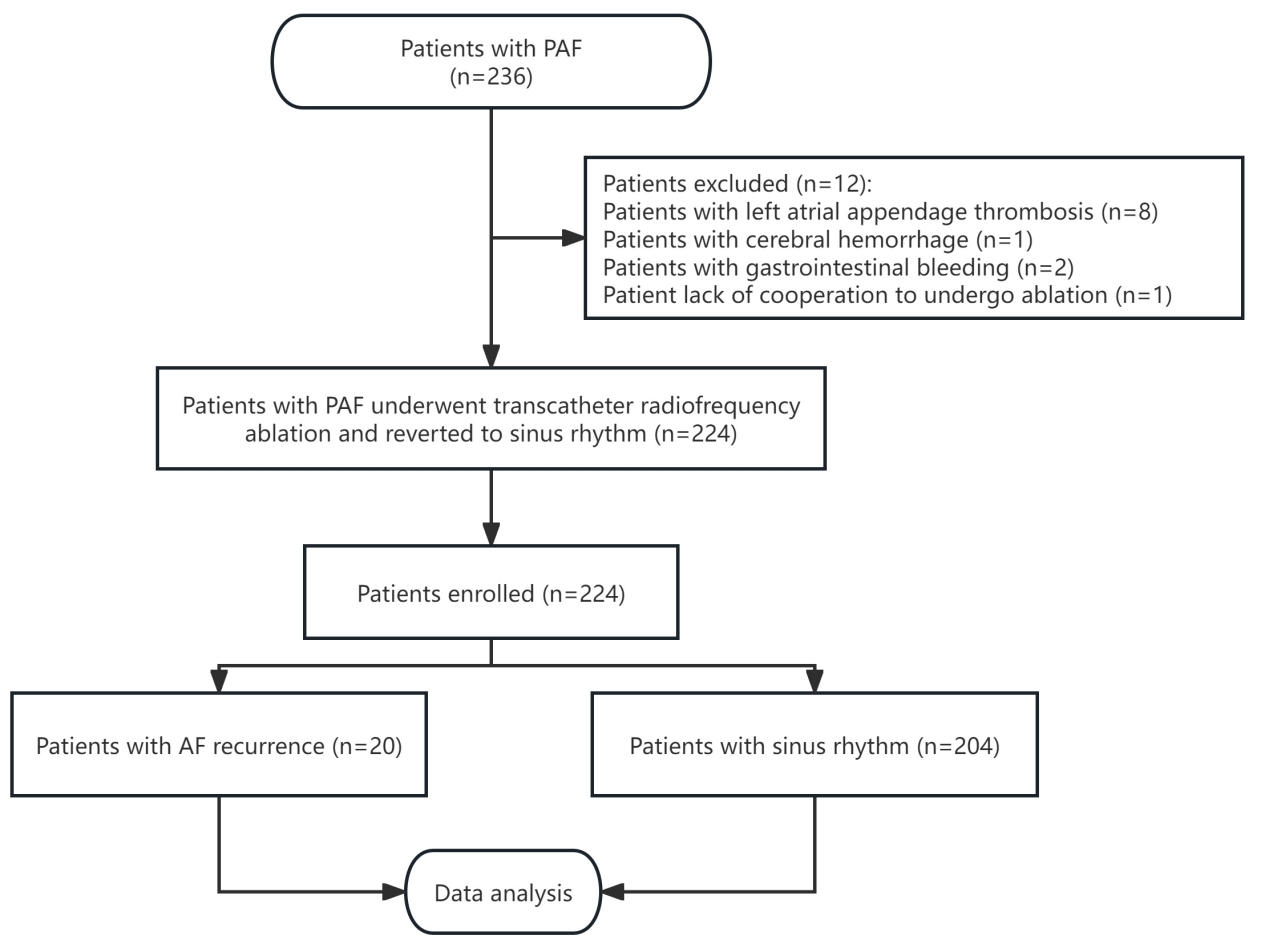
**Flowchart of the study**. PAF, paroxysmal atrial fibrillation; AF, atrial fibrillation.

According to the inclusion criteria, 236 patients diagnosed with PAF were enrolled in this study. Among the initial participants, eight patients were found with left atrial appendage thrombosis, three experienced severe bleeding events (one case of cerebral hemorrhage and two cases of gastrointestinal bleeding), and one was unable to undergo RFA due to a lack of cooperation. Hence, these cases were excluded from the final analysis. Consequently, 224 patients underwent RFA and successfully reverted to sinus rhythm (Fig. 1). This group comprised 129 males and 95 females, averaging 61.500 ± 9.210 years. Based on the preoperative AHR in sinus rhythm state, the patients were divided into three groups: 75 patients in the low heart rate group (<70 bpm), 74 patients in the medium heart rate group (70–75 bpm), and 75 patients in the high heart rate group (>75 bpm). After 3–6 months of follow-up, 20 patients (8.929%) experienced recurrence of AF, including 11 patients in the low heart rate group, six in the medium heart rate group, and three in the high heart rate group. There were statistically significant differences (*p* < 0.05) in the AHR, LAD, and history of peripheral vascular disease among the three groups of PAF patients. However, no statistically significant differences were observed in other indicators. Furthermore, as the AHR increased from the low to the high level, the recurrence rate of AF gradually decreased from 14.667% in the low heart rate group to 8.108% in the medium heart rate group and finally to 4.000% in the high heart rate group (*p* = 0.069, Table 1).

**Table 1.** **Comparison of clinical parameters, dynamic electrocardiogram, and echocardiographic indicators with different AHR levels in PAF patients**.

AHR tertiles (bpm)		Total	Low	Medium	High	*p*-value
			<70	70–75	>75	
N (cases)		224.000	75.000	74.000	75.000	
Female, n (%)		95 (42.411)	35 (46.667)	30 (40.541)	30 (40.000)	0.657
Age (years)		61.500 ± 9.210	62.707 ± 7.122	60.838 ± 10.345	60.947 ± 9.853	0.381
BMI (kg/m^2^)		24.569 ± 3.352	24.589 ± 3.367	24.483 ± 2.963	24.633 ± 3.721	0.962
AF duration (months)		6.834 ± 2.456	6.113 ± 2.572	6.985 ± 2.387	6.754 ± 2.076	0.066
BUN (mmol/L)		5.290 ± 1.466	5.305 ± 1.388	5.420 ± 1.556	5.145 ± 1.459	0.518
TC (mmol/L)		4.138 ± 0.985	3.945 ± 1.127	4.196 ± 0.805	4.274 ± 0.976	0.103
AHR (bpm)		72.857 ± 11.061	61.520 ± 4.598	72.486 ± 2.360	84.560 ± 8.535	<0.001
Box-Cox transform (Total time of AF)		0.550 ± 0.013	0.547 ± 0.010	0.550 ± 0.013	0.552 ± 0.015	0.058
Box-Cox transform (Total episodes of AF)		5.411 ± 1.000	5.231 ± 0.802	5.449 ± 1.049	5.553 ± 1.109	0.080
Box-Cox transform (Proportion of time in AF)		5.406 ± 1.000	5.234 ± 0.800	5.398 ± 0.989	5.587 ± 1.160	0.058
LAD (mm)		39.342 ± 5.813	40.627 ± 4.661	39.095 ± 6.411	38.274 ± 6.065	0.043
LVEDD (mm)		48.977 ± 4.714	49.213 ± 4.313	49.351 ± 5.140	48.356 ± 4.659	0.384
LVESD (mm)		32.495 ± 4.078	32.520 ± 3.338	32.878 ± 4.885	32.082 ± 3.883	0.497
LVEF (%)		61.338 ± 5.053	61.453 ± 4.431	60.892 ± 6.236	61.671 ± 4.295	0.629
Comorbidity, n (%)						
	Hypertension, n (%)	135 (60.268)	49 (65.333)	41 (55.405)	45 (60.000)	0.464
	Diabetes mellitus, n (%)	25 (11.161)	11 (14.667)	8 (10.811)	6 (8.000)	0.429
	Coronary heart disease, n (%)	29 (12.946)	11 (14.667)	6 (8.108)	12 (16.000)	0.308
	Hyperlipidemia, n (%)	36 (16.071)	12 (16.000)	10 (13.514)	14 (18.667)	0.693
	History of stroke or TIA, n (%)	21 (9.375)	11 (14.667)	5 (6.757)	5 (6.667)	0.156
	Peripheral vascular disease, n (%)	68 (30.357)	26 (34.667)	28 (37.838)	14 (18.667)	0.024
CHA2DS2-VASc						0.985
	0	39 (17.411)	14 (18.667)	12 (16.216)	13 (17.333)	
	1	78 (34.821)	27 (36.000)	25 (33.784)	26 (34.667)	
	≥2	105 (46.875)	34 (45.333)	37 (50.000)	34 (45.333)	
Smoking, n (%)		49 (21.875)	14 (18.667)	19 (25.676)	16 (21.333)	0.580
Drinking, n (%)		30 (13.393)	12 (16.000)	9 (12.162)	9 (12.000)	0.718
Antiarrhythmic drugs, n (%)						0.304
	Amiodarone	35 (15.625)	11 (14.667)	11 (14.865)	13 (17.333)	
	Dronedarone	14 (6.250)	5 (6.667)	4 (5.405)	5 (6.667)	
	Propafenone	15 (6.696)	4 (5.333)	5 (6.757)	6 (8.000)	
	Sotalol	20 (8.929)	6 (8.000)	7 (9.459)	7 (9.333)	
	β-receptor blockers	60 (26.786)	18 (24.000)	20 (27.027)	22 (29.333)	
AF recurrence, n (%)		20 (8.929)	11 (14.667)	6 (8.108)	3 (4.000)	0.069

Abbreviations: AHR, average heart rate; BMI, body mass index; BUN, blood–urea–nitrogen; TC, total cholesterol; AF, atrial fibrillation; LAD, left atrial diameter; LVEDD, left ventricular end-diastolic dimension; LVESD, left ventricular end-systolic dimension; LVEF, left ventricular ejection fraction; TIA, transient ischemic attack; CHA2DS2-VASc, stroke risk score of AF patients; PAF, paroxysmal atrial fibrillation.

### 3.2 Univariate Logistic Regression Analysis of Clinical Parameters, Dynamic Electrocardiogram, and Echocardiographic Indicators and AF Recurrence

Using AF recurrence after RFA as the dependent variable and clinical parameters and echocardiographic and dynamic electrocardiogram indicators, including AHR, as the independent variables, a univariate logistic regression analysis was performed to identify potential factors associated with AF recurrence. The result showed that total time of AF, total episodes of AF, proportion of time in AF, AHR in sinus rhythm state, LVEDD, LVESD, and LVEF were potential factors related to AF recurrence (*p* < 0.10) (Table 2).

**Table 2.** **Univariate logistic regression analysis of clinical parameters, dynamic electrocardiogram and echocardiographic indicators, and AF recurrence**.

Covariate		Statistics	OR	*p*-value
Female, n (%)		95 (42.411)	1.123 (0.446, 2.828)	0.806
Age (years)		61.500 ± 9.210	0.999 (0.951, 1.050)	0.980
BMI (kg/m^2^)		24.569 ± 3.352	1.075 (0.944, 1.223)	0.276
AF duration (months)		6.834 ± 2.456	1.059 (0.971, 1.134)	0.776
BUN (mmol/L)		5.290 ± 1.466	0.898 (0.644, 1.252)	0.525
TC (mmol/L)		4.138 ± 0.985	1.067 (0.669, 1.703)	0.785
AHR (bpm		72.857 ± 11.061	0.957 (0.913, 1.004)	0.073
Box-Cox transform (Total time of AF)		0.550 ± 0.013	inf. (inf., inf.)	<0.000
Box-Cox transform (Total episodes of AF)		5.411 ± 1.000	2.587 (1.822, 3.673)	<0.000
Box-Cox transform (Proportion of time in AF)		5.406 ± 1.000	2.501 (1.765, 3.545)	<0.000
LAD (mm)		39.342 ± 5.813	1.058 (0.978, 1.145)	0.158
LVEDD (mm)		48.977 ± 4.714	1.131 (1.029, 1.243)	0.011
LVESD (mm)		32.495 ± 4.078	1.180 (1.070, 1.302)	0.001
LVEF (%)		61.338 ± 5.053	0.859 (0.791, 0.933)	0.000
Comorbidity, n (%)				
	Hypertension, n (%)	135 (60.268)	0.632 (0.252, 1.587)	0.329
	Diabetes mellitus, n (%)	25 (11.161)	1.460 (0.396, 5.382)	0.570
	Coronary heart disease, n (%)	29 (12.946)	1.208 (0.331, 4.409)	0.775
	Hyperlipidemia, n (%)	36 (16.071)	0.914 (0.254, 3.298)	0.891
	History of stroke or TIA, n (%)	21 (9.375)	0.000 (0.000, Inf.)	0.991
	Peripheral vascular disease, n (%)	68 (30.357)	0.547 (0.176, 1.701)	0.297
CHA2DS2-VASc				
	0	39 (17.411)	Reference	
	1	78 (34.821)	1.043 (0.956, 1.142)	0.419
	≥2	105 (46.875)	1.114 (0.933, 1.14)	0.657
Smoking, n (%)		49 (21.875)	0.606 (0.170, 2.160)	0.440
Drinking, n (%)		30 (13.393)	0.318 (0.041, 2.465)	0.273
Antiarrhythmic drugs, n (%)				
	Amiodarone	35 (15.625)	0.949 (0.263, 3.426)	0.936
	Dronedarone	14 (6.250)	0.773 (0.096, 6.239)	0.809
	Propafenone	15 (6.696)	1.632 (0.341, 7.809)	0.539
	Sotalol	20 (8.929)	1.148 (0.246, 5.350)	0.860
	β-receptor blockers	60 (26.786)	0.661 (0.212, 2.062)	0.475

Abbreviations: BMI, body mass index; BUN, blood–urea–nitrogen; TC, total cholesterol; AHR, average heart rate; AF, atrial fibrillation; LAD, left atrial diameter; LVEDD, left ventricular end-diastolic dimension; LVESD, left ventricular end-systolic dimension; LVEF, left ventricular ejection fraction; TIA, transient ischemic attack; CHA2DS2-VASc, stroke risk score of AF patients; OR, odds ratio; inf. (inf., inf.), it is suggested that the coefficient estimate corresponding to Box-Cox transform (total time of AF) tends to positive infinity, the OR is going to go to infinity.

### 3.3 Multivariate Logistic Regression Analysis of the Effect of AHR on AF Recurrence

Table 3 presented both univariate and multivariate logistic regression analysis outcomes, representing the AHR in the sinus rhythm state expressed as continuous variables and three tertiles, respectively. Model 0 corresponded to the unadjusted covariate equation, equivalent to the univariate logistic regression analysis. Model I was the preliminary adjusted covariate equation, which included adjustments for seven covariates: age, gender, BMI, peripheral vascular disease, LVEF, LVEDD, and total episodes of AF. Model II was the fully adjusted covariate equation, which included adjustments for nine covariates: age, gender, BMI, peripheral vascular disease, LVEF, LVEDD, total episodes of AF, total time of AF, and proportion of time in AF.

**Table 3.** **Multivariate logistic regression analysis of the effect of AHR on AF recurrence**.

Variable		Model 0		Model I		Model II	
		OR (95% CI)	*p*-value	OR (95% CI)	*p*-value	OR (95% CI)	*p*-value
AHR		0.957 (0.913, 1.004)	0.073	0.929 (0.875, 0.987)	0.017	0.849 (0.729, 0.988)	0.035
AHR tertiles							
	Low	Reference		Reference		Reference	
	Medium	0.513 (0.179, 1.469)	0.214	0.254 (0.066, 0.975)	0.046	0.121 (0.015, 0.982)	0.048
	High	0.242 (0.065, 0.908)	0.035	0.099 (0.017, 0.559)	0.009	0.025 (0.001, 0.742)	0.033

Abbreviations: CI, confidence interval; AHR, average heart rate; BMI, body mass index; AF, atrial fibrillation; LVEDD, left ventricular end-diastolic dimension; LVEF, left ventricular ejection fraction; OR, odds ratio. Model I adjusted for gender, age, BMI, peripheral vascular disease, LVEDD, LVEF, and total episodes of AF. Model II adjusted for gender, age, BMI, peripheral vascular disease, LVEDD, LVEF, total time of AF, total episodes of AF, and proportion of time in AF.

When AHR in a sinus rhythm state was treated as a continuous variable, the regression equations of Model I and Model II demonstrated that an increased preoperative AHR in patients with AF decreased the risk of recurrence after RFA. These findings were statistically significant in both models (odds ratio (OR): 0.929, 95% confidence interval (CI): 0.875–0.987, *p* = 0.017; OR: 0.849, 95% CI: 0.729–0.988, *p* = 0.035; respectively).

Setting AHR in sinus rhythm state into three tertiles, we also observed a trend of increasing AHR being associated with a decreasing risk of AF recurrence in Model 0, Model I, and Model II regression equations (OR: 0.242, 95% CI: 0.065–0.908, *p* = 0.035; OR: 0.099, 95% CI: 0.017–0.559, *p* = 0.009; and OR: 0.025, 95% CI: 0.001–0.742, *p* = 0.033; respectively).

### 3.4 Curve Fitting

**Fig. 2.**
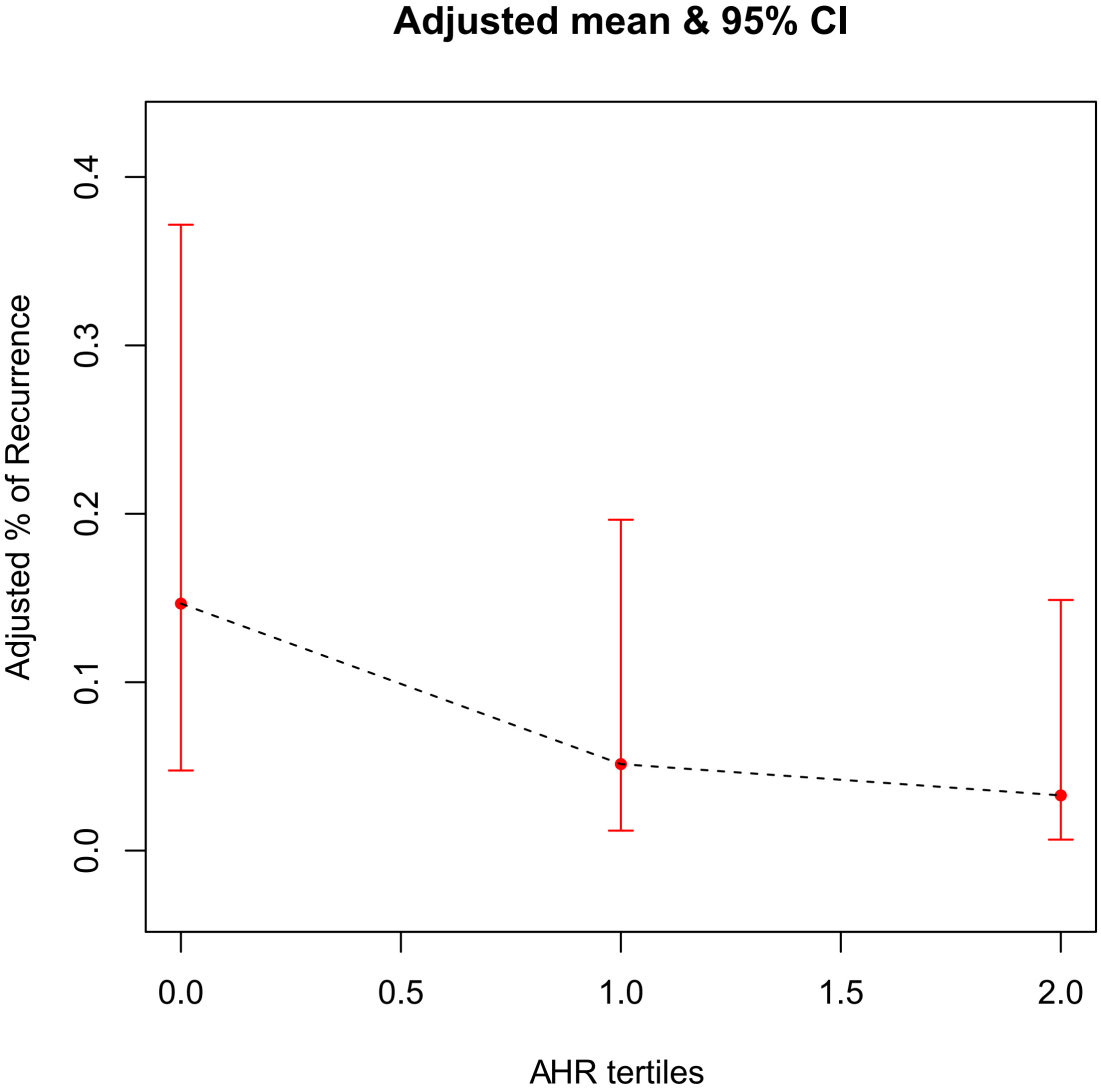
**The association between AHR tertiles and AF recurrence risk**. The black dotted line represents the fitted line between the recurrence rate of AF and the AHR tertiles, and the red line is the 95% confidence interval. AHR, average heart rate; AF, atrial fibrillation.

A generalized additive model was used to test the correlation between AHR and AF recurrence. The finding revealed that, in the AHR tertiles groups, after being fully adjusted for covariates such as age, gender, BMI, peripheral vascular disease, LVEF, LVEDD, total episodes of AF, total time of AF and proportion of time in AF, there was a gradual decrease in the probability of postoperative AF recurrence as the grouping level increased. The two variables were approximately linearly and negatively associated (*p* < 0.05, Fig. 2).

## 4. Discussion

The findings of this study revealed that for patients with PAF, the recurrence rate of AF decreased with an increase in the AHR in the sinus rhythm state after achieving ventricular rate control before RFA.

In recent years, the management of AF has undergone revolutionary changes. Transcatheter RFA has become a first-line treatment option for drug-resistant and symptomatic PAF patients [5]. However, the high rate of AF recurrence after RFA often diminishes the benefits of ablation. Therefore, the early identification of risk factors for AF recurrence is crucial for detecting and treating such arrhythmias to prevent complications such as stroke.

The guidelines from the American Heart Association [28] suggest long-term administration of a β-receptor blocker or ND-CCB to control ventricular rate in patients with PAF and achieve the resting heart rate goal of <80 bpm, to enhance clinical symptoms, preserve cardiac function, improve quality of life and prognosis. Nonetheless, the guidelines do not address the potential influence of a low resting heart rate on the onset of AF and the recurrence of AF after ablation.

Several scholars have researched the relationship between heart rate and the development or recurrence of AF and found that a low heart rate can increase the risk of both AF occurrence and postoperative recurrence. For example, O’Neal *et al*. [29] showed that a decreased resting heart rate is associated with an increased risk of AF, and this finding is consistent in subgroups of age, gender, race, and coronary atherosclerotic heart disease. Other two large independent cohort studies, namely the Copenhagen Electrocardiographic Study [30] and the Tromsø Study [22], have discovered that a low resting heart rate (<50 bpm) is an independent predictor of AF. Furthermore, Goff *et al*. [31] demonstrated a negative correlation between an increase in postoperative resting heart rate and the recurrence of AF one year after ablation, highlighting that patients with a postoperative resting heart rate increase of more than 15 bpm have a reduced risk of AF recurrence. Another study [24], including patients with paroxysmal AF and persistent AF, likewise concluded that an increase in resting heart rate after RFA is negatively associated with AF recurrence. Yu *et al*. [32] proposed that a high postoperative AHR is linked to a reduced risk of AF recurrence, and a high preoperative AHR is one of the strongest predictive factors for a high postoperative AHR. Wu *et al*. [23] found that a preoperative low resting heart rate (<50 bpm) is an independent predictor of postoperative recurrence in patients with PAF who are 65 years or older. von Olshausen *et al*. [33] confirmed that patients with a sinus heart rate change <11 bpm (pre-ablation to 3 months post-ablation) were at higher risk of recurrences during one-year post-ablation. Vassallo *et al*. [34] stated that AF ablation with high-power–short-duration incidental cardiac parasympathetic denervation identified that patients with lower heart rate increase are prone to recurrence, whereas those with higher heart rate increase had higher maintenance of sinus rhythm at a long-term follow-up. The results of this study aligned with previous research.

As previously mentioned, several studies demonstrated that patients with significantly increasing AHR post-ablation had a lower recurrence rate of AF. The cardiac autonomic nervous system (ANS) plays an important role in the pathophysiology of AF [5]. The intrinsic part of the ANS is located primarily in the ganglionated plexus (GP) [35], which primarily contains parasympathetic but also sympathetic neurons [36], considered to influence the sinus rate, atrial refractory period and atrioventricular conduction [37]. Most of the GP lie near the pulmonary vein ostia and left atrium junction [38, 39], where pulmonary vein isolation is operated. Therefore, inadvertent ablation of the GP is possible, which then contributes to the increase in post-ablation heart rate through parasympathetic denervation and could ultimately be relative to a lower recurrence rate of AF.

In this study, we demonstrated that the AHR in the preoperative sinus rhythm state in patients with PAF was negatively associated with the risk of postoperative AF recurrence, which various biological mechanisms can explain. Firstly, it was observed that patients with lower AHR had significantly larger LAD compared to those with higher AHR. This can be attributed to left atrial enlargement, which promotes electrical and anatomical remodeling of the left atrium and leads to the secretion of inflammatory factors, subsequent fibrosis, left atrial matrix remodeling [40], increased P-wave dispersion [41], prolongation of intra-atrial and inter-atrial conduction times [42], and significant atrioventricular block.All these changes increase sensitivity to AF. Secondly, maintaining sinus rhythm for at least three months after ablation is crucial for electrical and anatomical reverse remodeling of the left atrium. However, in patients with low preoperative AHR, the routine use of AAD becomes contraindicated, or the dosage is limited after ablation. This may result in difficulty maintaining sinus heart rate and delayed reverse remodeling of the left atrium, which can easily result in AF recurrence. Lastly, a low resting heart rate is associated with ANS activity or subclinical sinoatrial node dysfunction [29, 32]. On the one hand, the ANS activity is an important modulating factor in the perpetuation of AF [43]. Increased ANS activity shortens the duration of the action potential by increasing acetylcholine-dependent potassium current while also leading to calcium transients by increasing norepinephrine secretion [44, 45]. The above two factors jointly increase early post-depolarization potential, contributing to the formation of AF trigger potential [46] and subsequently increasing the recurrence rate of AF. Meanwhile, lower AHR could be an indicator of increased ANS activity, which may make successful GP ablation more difficult, while the addition of GP ablation has been confirmed to be related to reduced arrhythmia recurrence in PAF patients in a meta-analysis [47]. On the other hand, subclinical sinus node dysfunction is related to sinoatrial node dysfunction and age-related changes and fibrosis in the cardiac conduction system outside the atrial myocardium and sinoatrial node. These anatomical changes create a substrate for the development of AF. Additionally, the accompanying bradycardia further facilitates AF development by increasing the probability of atrial arrhythmias and dispersion of refractory periods [48].

Limitations: Firstly, this study was a retrospective cohort study conducted at a single center. The sample size was relatively small, and the follow-up period was short. Secondly, the multivariate logistic regression model included several potential confounding factors that could influence the development of AF. However, as with other epidemiological studies, residual confounding factors were still possible. For instance, our model did not fully adjust for the duration and severity of certain conditions, such as hypertension, diabetes, and blood pressure and blood sugar control. Moreover, previous studies have shown that less postoperative heart rate increases compared to preoperation may be associated with higher postoperative AF recurrence [24, 31, 33, 34]; we will incorporate postoperative heart rate change parameters in the future to explore the risk of AF recurrence and possible mechanisms. Furthermore, the relationship between left atrial size and AF recurrence has been proved by several studies. In contrast, this study stated that left atrial size had no significant correlation with the recurrence of AF; this result may be relevant to the small sample and less left atrial remodeling in patients with PAF. In future research, we will expand the samples’ quantity and refine the evaluation of left atrial by adding left atrial volume and left atrial strain parameters to better explore the relationship between left atrial and AF recurrence. Finally, the mechanism underlying the occurrence of low preoperative AHR and postoperative recurrence in AF patients is currently unknown. Therefore, large-scale, multicenter, prospective case–control trials are still required to establish the correlation and mechanism between preoperative AHR and postoperative recurrence in patients with PAF.

## 5. Conclusions

In summary, the correlation between preoperative AHR in sinus rhythm state and the likelihood of postoperative AF recurrence offers valuable insights for risk stratification and clinical management of AF. Moreover, it serves as scientific evidence supporting the adjustment of preoperative AAD, intraoperative additional ablation of GP, and the identification and early intervention of high-risk patients prone to postoperative recurrence. In the case of AF patients with a low preoperative AHR, it is necessary to administer AAD with caution and to perform additional ablation of GP during RFA.

## Availability of Data and Materials

The datasets used and/or analyzed during the current study are available from the corresponding author on reasonable request.
